# Non-weight-bearing neural control of a powered transfemoral prosthesis

**DOI:** 10.1186/1743-0003-10-62

**Published:** 2013-06-19

**Authors:** Levi J Hargrove, Ann M Simon, Robert Lipschutz, Suzanne B Finucane, Todd A Kuiken

**Affiliations:** 1 Center for Bionic Medicine, Rehabilitation Institute of Chicago, Chicago, IL, USA; 2 Department of Physical Medicine and Rehabilitation, Northwestern University, Chicago, IL, USA; 3 Biomedical Engineering Department, Northwestern University, Evanston, IL, USA

## Abstract

Lower limb prostheses have traditionally been mechanically passive devices without electronic control systems. Microprocessor-controlled passive and powered devices have recently received much interest from the clinical and research communities. The control systems for these devices typically use finite-state controllers to interpret data measured from mechanical sensors embedded within the prosthesis. In this paper we investigated a control system that relied on information extracted from myoelectric signals to control a lower limb prosthesis while amputee patients were seated. Sagittal plane motions of the knee and ankle can be accurately (>90%) recognized and controlled in both a virtual environment and on an actuated transfemoral prosthesis using only myoelectric signals measured from nine residual thigh muscles. Patients also demonstrated accurate (~90%) control of both the femoral and tibial rotation degrees of freedom within the virtual environment. A channel subset investigation was completed and the results showed that only five residual thigh muscles are required to achieve accurate control. This research is the first step in our long-term goal of implementing myoelectric control of lower limb prostheses during both weight-bearing and non-weight-bearing activities for individuals with transfemoral amputation.

## Background

Lower limb amputation is a major cause of disability for millions of people worldwide. It is estimated that there are over 600,000 major lower limb amputees – transfemoral, transtibial, or hip disarticulated patients – living in the United States [1]. Individuals with a transfemoral amputation comprise approximately half of the major lower limb amputee population and the majority of amputations are caused by dysvascular disease. Transfemoral amputations are often treated most effectively through use of a prosthetic leg.

There are three broad categories of prosthetic knees: 1) mechanically passive legs, 2) microprocessor-controlled mechanically passive legs, and 3) microprocessor-controlled mechanically active devices. Mechanically passive legs are readily available from a number of manufacturers and vary in costs and design complexity. In such devices, the movement of the prosthetic joint(s) relies on the properties of its mechanical components, such as hydraulic valves, linkage systems, pneumatic valves, or sliding joints. Prosthesis users must make compensatory movements with their trunk, pelvis, and residual limb to control the prosthesis; however, no sensors are required. Microprocessor-controlled passive prostheses employ sensors and a microcomputer for closed-loop control [2-4]. A finite-state controller receives kinematic and force information from the sensors attached to the prosthesis, detects the gait phase, and adjusts the mechanical impedance of the knee joint through a hydraulic damper [5] or by modifying a magnetic field [6]. The desired joint impedance is predetermined in each gait phase based on normal gait studies. Studies show that compared to the conventional passive prosthesis, the computerized prosthesis with varied impedance allows reduced energy consumption, improved smoothness of gait, and decreased hip work production during locomotion [7-9]. Neither mechanically passive, nor microprocessor-controlled passive prosthetic legs can generate positive power. This significantly impairs the ability of these prostheses to restore many natural locomotive functions, including ascending stairs and slopes and walking backwards, all of which require significant net positive power at the knee joint, ankle joint, or both [10-15]. Furthermore, mechanically passive prostheses cannot be automatically repositioned during non-weight-bearing situations; they need to be manually manipulated by the user.

Microprocessor-controlled, mechanically active prosthetic legs have recently become commercially available, and several additional prototypes are in various stages of development [16-19]. These devices use a similar high level control strategy to microprocessor-controlled mechanically passive prostheses; high-level state-based controllers interpret signals recorded from mechanical sensors embedded in the prosthesis or from an orthotic placed on the sound limb. The current state of the device then provides control information to lower-level position, force, torque, or impedance controllers to ensure that the actuator behaves properly. The capability of generating positive mechanical power greatly increases the number of locomotion modes which may be restored to the patient; however, improvements to the control system are required before this potential can be realized.

Surface electromyographic (EMG) signals may be decoded to provide an estimate of neural activation and have been used to extract control information for upper limb prostheses for many decades. EMG signals have also been investigated for use with intent recognition for microprocessor-controlled above and below knee prostheses [20-27]. Huang has shown that surface EMG signals can be decoded from transfemoral amputees to predict up to seven locomotion modes with classification accuracies of approximately 80%-95% [24]. Furthermore, it was also found that information extracted from EMG signals were complementary to information extracted from mechanical sensors. Simple sensor fusion improved the accuracy and responsiveness of mode recognition [25].

One particular mode which seems to be ideally suited for EMG control is during non-weight-bearing situations. The user may wish to reposition their prosthesis to help dress themselves or prepare for transfers. Researchers have previously proposed using EMG pattern recognition to volitionally control knee movements for a transfemoral prosthesis and noted high classification accuracies for knee flexion and extension [28,29]. Other signal processing techniques such as Kalman filtering have also been proposed to track knee movements using surface EMG signals measured from the thigh muscles[30]. These previous studies were completed using healthy control subjects and did not consider controlling ankle movements. Ha et al. recently showed that EMG signals from the quadriceps and hamstrings could be reliably decoded using quadratic discriminant analysis and used with a knee impedance paradigm to control an amputee’s knee position [22]. This work showed that patients could accurately track a position command in a virtual environment but it was not implemented on a powered prosthesis. We recently completed preliminary research which showed that both knee and ankle movements could be accurately decoded using EMG signals measured from the residual thigh muscles of transfemoral amputees [26]. That work was limited in that it was only completed with four subjects in a virtual environment. In this contribution, we extend our previous research by considering additional amputee patients and demonstrating that the technique can be used to control a powered knee prosthesis.

## Methods

**Figure 1:**
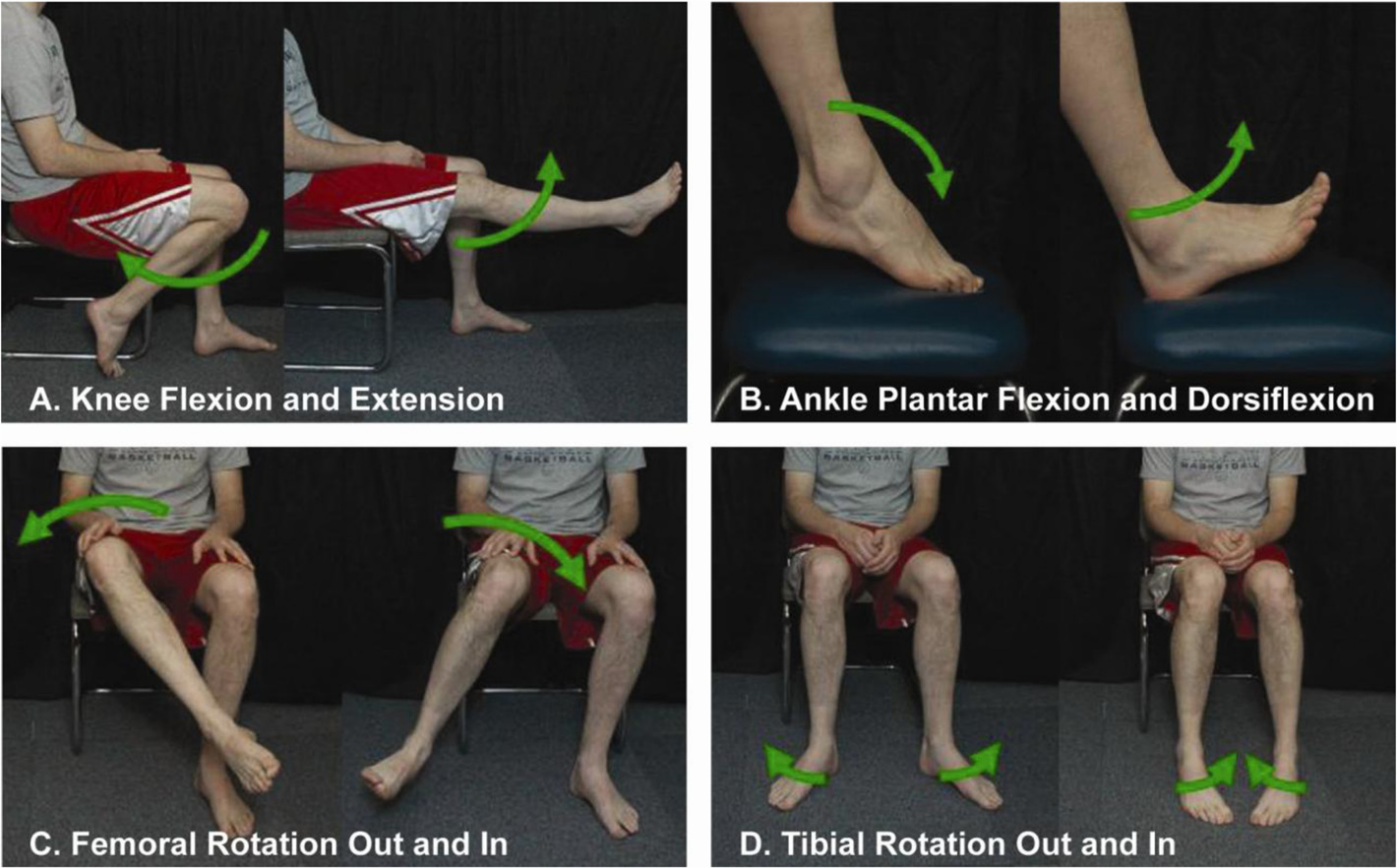
Photographs depicting the trained motions including A) knee flexion and extension, B) ankle plantar flexion and dorsiflexion, C) femoral rotation, D) and tibial rotation.

Two sets of experiments were completed between September 2009 and Sept 2011 at the Rehabilitation Institute of Chicago. The Northwestern University Institutional Review Board approved the studies, and written informed consent was obtained from all study subjects. Each experiment had two components. The first component comprised an offline data collection procedure from which the pattern recognition system was trained and offline classification accuracy of the system was computed. During this component, the patient was provided no real-time feedback and attempted to move their phantom limb as instructed by a photograph (Figure 1) displayed on a computer screen. The second component comprised a real-time control experiment, called the motion test, in which the patient controlled a virtual (Experiment 1) or a physical (Experiment 2) prosthesis in real time.

### A. Experiment 1: Non-weight-bearing Control in a Virtual Environment

**Figure 2:**
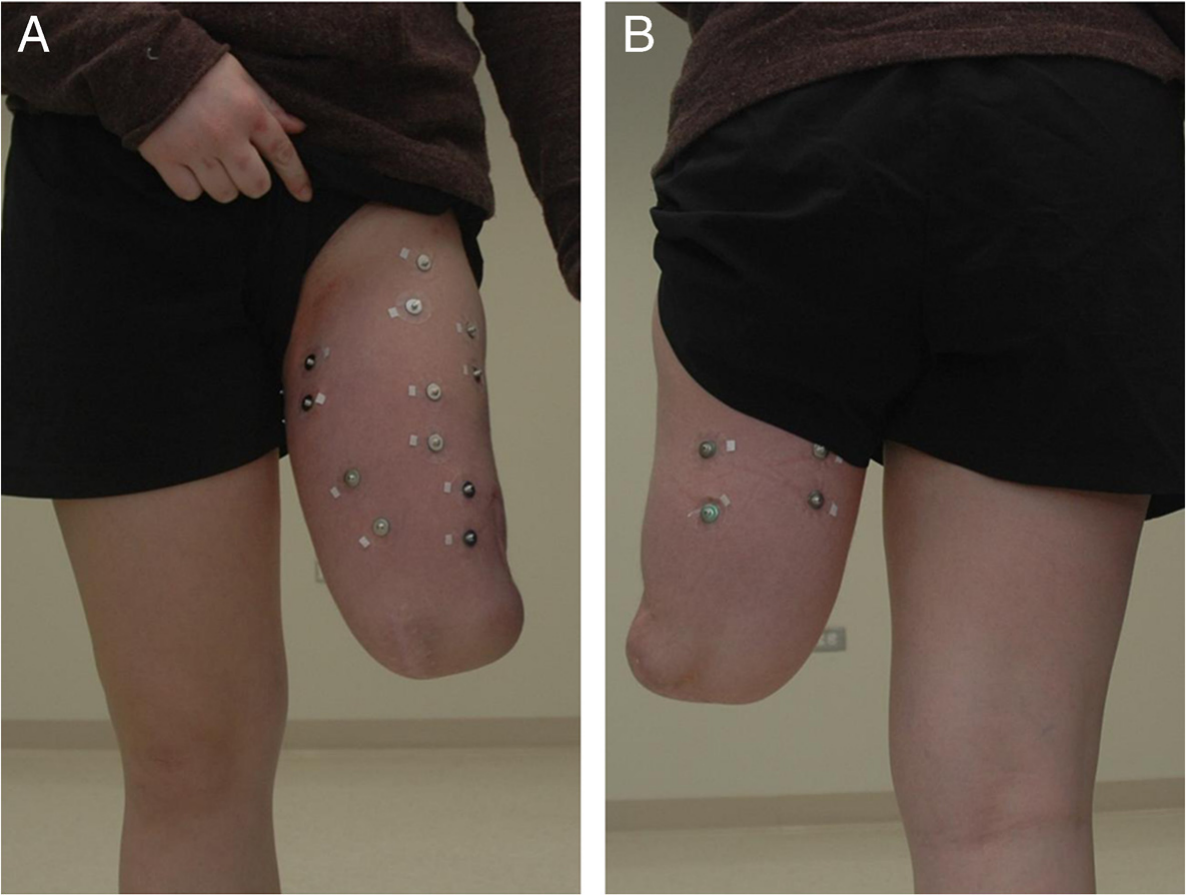
Photographs depicting the electrode placement on the patient’s A) anterior and B) posterior residual limb.

The data collection procedure for the non-weight-bearing control within the virtual environment has been briefly described previously [26] but is more thoroughly described in the subsequent section. Six subjects with unilateral transfemoral amputations (3 males, 3 females, mean (SD) age 47.8 (15.9) years, number of years post amputation 26.3 (17.7) years) and six healthy control subjects participated in this study. Subjects were seated and the following nine muscles were identified based on anatomical location and palpation: semitendinosus, sartorius, tensor fasciae latae, adductor magnus, gracilis, vastus medialis, rectus femoris, vastus lateralis, and long head of the biceps femoris. Nine adhesive, gelled silver–silver chloride electrode pairs were placed over the muscles of interest with an interelectrode spacing of approximately 3 cm (Figure 2). All data were amplified by a factor of approximately 1000 using a Delsys Bagnoli-16 amplifier, digitized using a 16-bit analog to digital converter, and transferred over a controller area network (CAN) bus using the Prosthesis Device Control Protocol [31].

Prior to any data collection, a therapist helped each patient to imagine performing the following movements: knee flexion, knee extension, ankle plantar flexion, ankle dorsiflexion, femoral rotation, and tibial rotation (Figure 1). EMG signals were viewed in real-time to ensure that electrodes were making good contact with the skin and signal gain was set at an appropriate level. Custom software—Control Algorithms for Prosthetic Systems (CAPS)—instructed the subjects to perform the movements listed previously. Data were also collected for a relaxation state which corresponded to a no movement command. The order that the trials were collected was not randomized for the collection of the classifier offline data and eight repetitions of 3 s each were collected for each motion. Data from offline repetitions 1–4 were used to train a pattern recognition system, and data from offline repetitions 5–8 were used to compute classification accuracy.

The pattern recognition system used was based on time-domain features extracted from 250 ms analysis windows and classified by a linear discriminant analysis classifier. Classifier decisions were made every 50 ms which allowed for 200 ms to be retained in an overlapping window scheme. This system has been used extensively and been shown to provide good classification performance for upper limb amputees [32]. Furthermore, this combination of feature-set and classifier has shown promise in recognizing ambulation modes during weight-bearing activities and performs equivalently to non-linear artificial neural network classifiers [24]. The algorithm and equations required for computing feature extraction and classification are provided in Appendix A.

The pattern recognition system was evaluated on its ability to provide high classification accuracy and real-time controllability. Classification accuracy was defined as the percentage of offline test data correctly predicted by the pattern recognition system trained with the offline training data. Real-time controllability was evaluated by the patient’s performance when completing a virtual environment motion test [32]. The motion test required subjects to replicate motions displayed on a computer screen while real-time position feedback was provided by a virtual environment avatar. Performance metrics for the motion test included motion completion time, motion completion percentage, and motion selection time [26]. Motion completion time was the elapsed time from movement onset until the subject successfully moved the virtual limb through the complete range of motion. The speed of the virtual limb was normalized such that the patient could move the prosthesis through the range of motion in a minimum time of 1 s when the classifier correctly classified the movement (i.e. corresponding to 20 correct decisions). The trial ended unsuccessfully if a patient could not move the virtual limb through the complete range of movement within 15 s. Motion completion percentage was the number of successfully completed motions divided by the total number of trials. Motion selection time was the elapsed time from movement onset to the first correct movement classification.

The offline training data were used to create two pattern recognition systems. The first pattern recognition system was trained to recognize a 2 degree of freedom (DOF) subset of all the collected movements: knee flexion/extension, ankle plantar flexion/dorsi-flexion and a no movement class. After the system was trained, the patient completed a real-time control test using feedback provided by the virtual limb. The motion test configuration consisted of nine real-time trials of each of the four movements (the no motion class was not tested) which were presented in random order. The second pattern recognition system was trained using the offline training data to recognize all movements. This motion test configuration consisted of 3 real-time trials of each of the eight movements which were presented in a random order.

An additional offline analysis was performed to determine the number of channels required to achieve acceptable pattern recognition accuracy for transfemoral amputees only. Exhaustive searches of the optimal channel subsets ranging from lengths 1–9 were made for the 2 and 4 DOF pattern recognition system [33]. Optimal channel subsets of length m were defined as the subset of channels that produced the highest classification accuracy when only m channels were used as input to the pattern recognition system. Additionally, the classification accuracies were evaluated when only data from the rectus femoris and semitendinosus were used as this configuration most resembled the EMG input to the two channel system described by Ha et al. [22]. Due to time constraints during the experiments, motion tests were not performed for different channel subset lengths.

### B. Experiment 2: Non-weight-bearing Control using a Physical Prosthesis

Three of the six transfemoral amputee participants returned to complete a second experiment to evaluate their performance when controlling a powered knee prosthesis. A prosthetist created a transparent test socket for each patient with stainless steel dome electrodes (Motion Control Inc.) embedded on the interior socket wall at the same electrode locations as Experiment 1. A male snap was threaded on the exterior socket wall to provide convenient connections to the same Bagnoli-16 EMG amplifier used in the previous experiment. Once again, data were amplified by a factor of 1000, sampled by a 16 bit analog-to-digital converter and streamed across a CAN bus to CAPS software.

**Figure 3:**
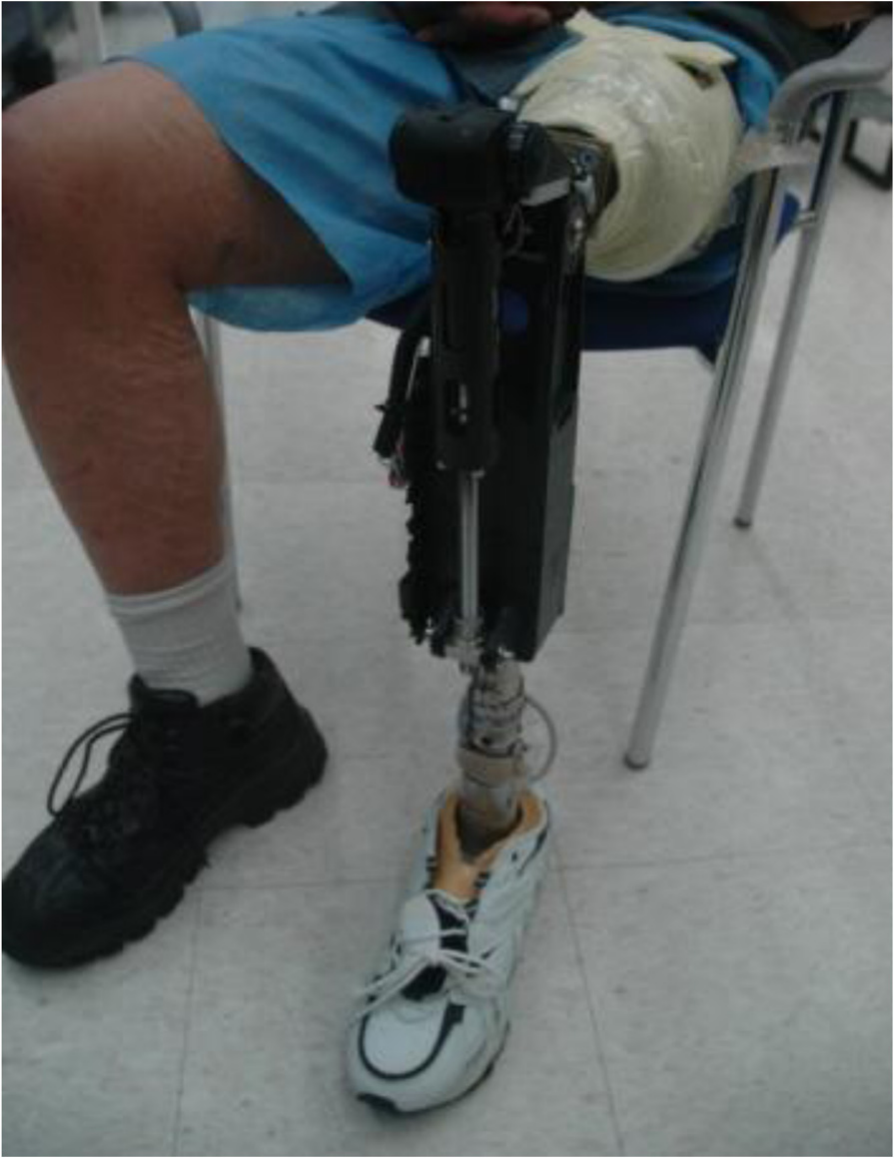
Transfemoral amputee wearing the powered knee prosthesis.

**Figure 4:**
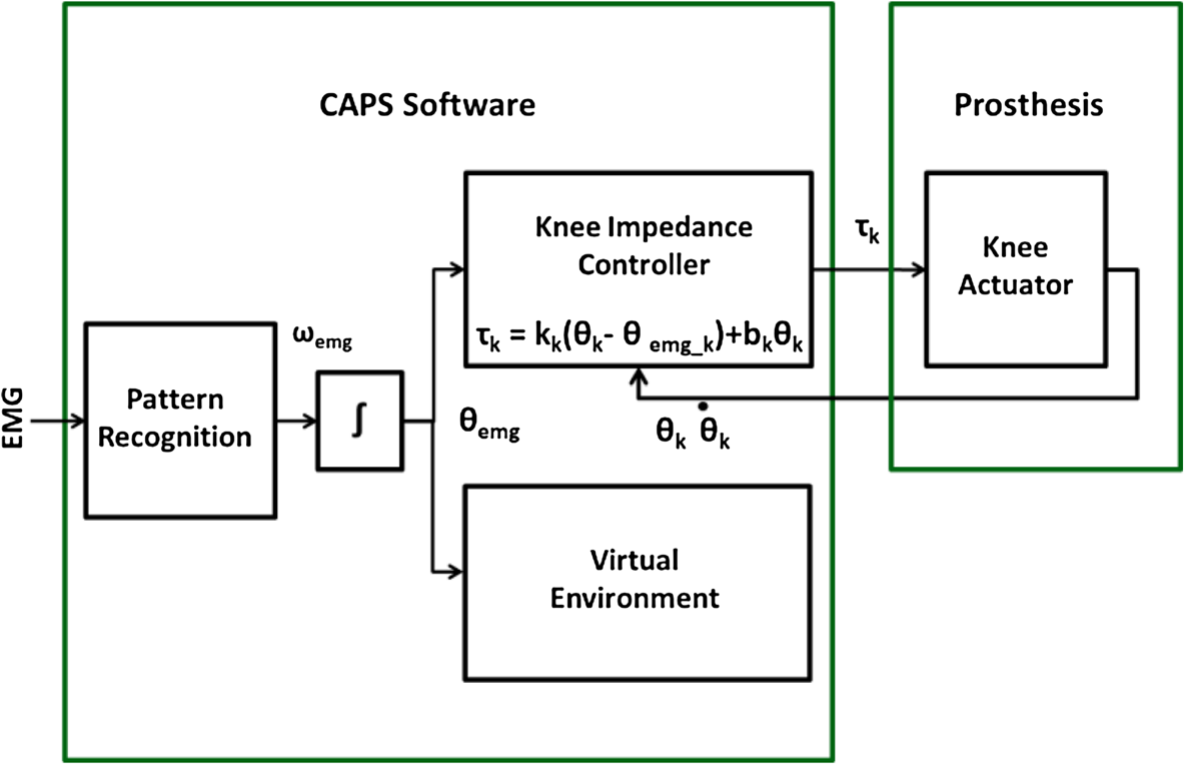
Architecture of the impedance controller used to generate the torque command provided to the powered knee prosthesis.

The powered knee prosthesis used in this experiment was designed and fabricated at Vanderbilt University and is similar to the prosthesis described in previous work [22,34] except that the ankle actuation unit was removed (Figure 3). The prosthesis weighed approximately 8 lbs, was battery powered, and the knee was actuated using a Maxon EC30 motor through a ball screw transmission. The knee was attached to a Seattle Lightfoot. A volitional impedance controller was created within CAPS (Figure 4) and was very similar to architecture described previously by the Vanderbilt Group. The pattern recognition system described in Experiment 1 provided the two mutually exclusive outputs ω_k_emg_ and ω_a_emg_, corresponding to knee and ankle velocities, respectively. These velocities were integrated to provide an estimate of the desired knee and ankle positions. A joint torque command was generated according to the following equation:

(1) $$\tau_{i}=k_{i}\left(\theta_{i}-\theta_{i_{\mathrm{emg}}}\right)+b_{i}\dot{\theta }$$

where *i* was an index corresponding to the knee or ankle, *k* was an empirically determined virtual stiffness, *θ* was the position measured from the prosthesis, θ_emg_ was an estimate of the desired joint position, *b* was an empirically determined virtual damping term, and $\dot{\theta }$ was the joint velocity measured from the prosthesis.

When the prosthesis was initially powered on, the tuning parameters (*k* and *b*) were set to 0 such that a joint torque command of 0 Nm was sent to the device while training and testing data were collected. The data were collected using the same procedure as used in Experiment 1 except only knee flexion, knee extension, ankle plantar flexion, and ankle dorsiflexion, and no motion data were collected. Next, the myoelectric impedance control parameters were tuned empirically. The values of *k*_*k*_ and *b*_*k*_ were slowly adjusted until the subject could move the knee through the full range of motion at a comfortable speed with a smooth kinematic profile. Since the prosthesis did not contain an ankle actuation unit, the ankle tuning parameters, *k*_*a*_ and *b*_*a*,_ remained at 0. These parameters would also need to be adjusted in order to control an ankle actuation unit. Subjects practiced controlling the knee for several minutes prior to completing motion tests with the physical prosthesis.

The motion tests were very similar to those described in Experiment 1 except that the order of motions was not randomized; knee flexion and extension were tested first. Subjects were cued by the experimenter to perform the appropriate motion and move the knee joint through the full range of motion. Ankle motion tests were completed with the prosthetic knee positioned at 90 degrees of knee flexion (i.e. neutral position when sitting) and at 45 degrees of knee flexion. Testing in the two different positions allowed us to determine if the pattern recognition system could still recognize ankle motions when the knee was repositioned. Feedback was provided to the subject by both the virtual environment and the physical prosthesis: the output of the pattern recognition classifier was displayed on a computer monitor and if the pattern recognition system erroneously decoded a knee command, then the prosthesis would move incorrectly. The performance metrics of the motion tests were motion completion percentage and motion completion time.

## Results

### A. Experiment 1: Non-weight-bearing Control in a Virtual Environment

**Figure 5:**
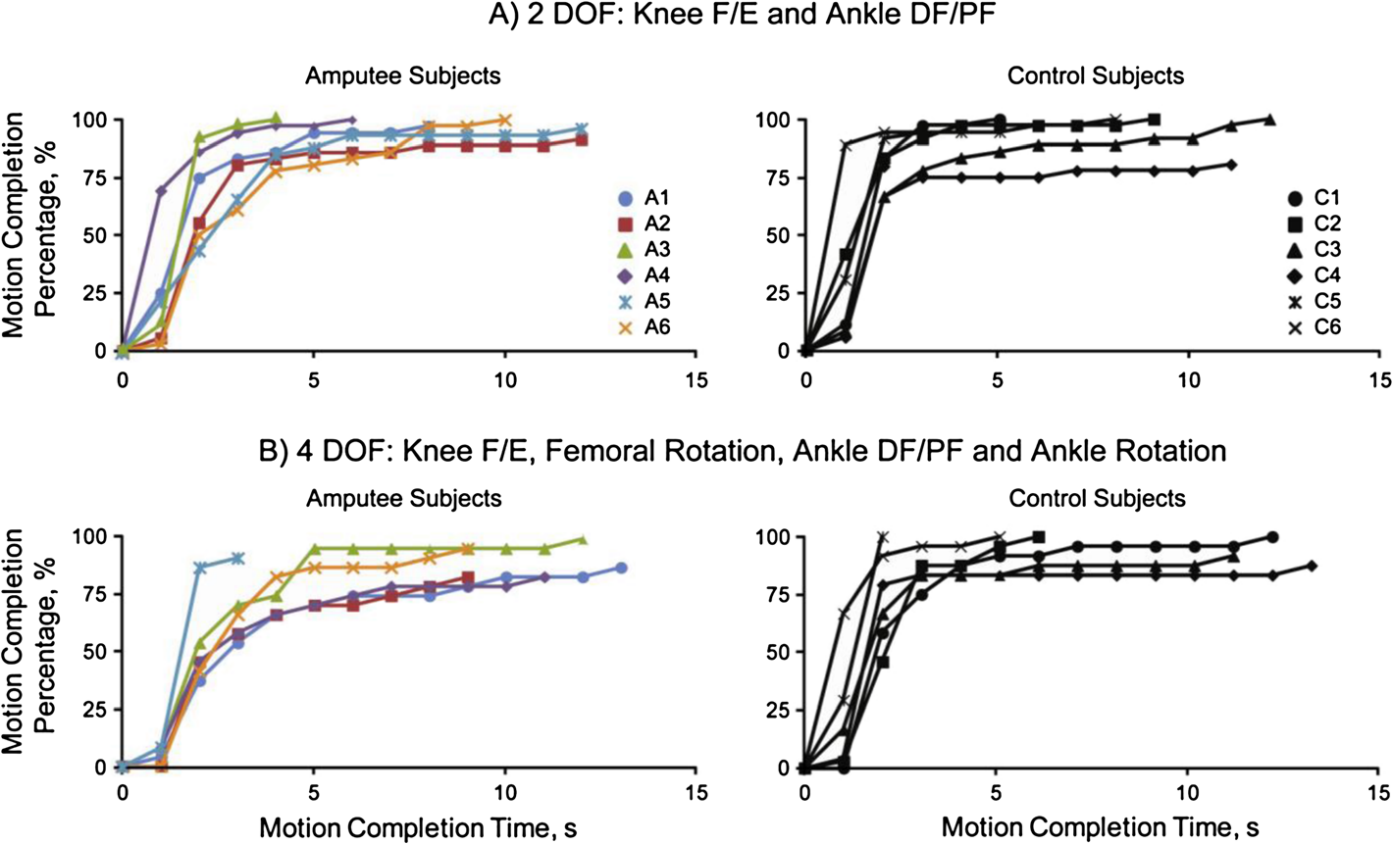
Motion completion percentage as a function of completion time for transfemoral amputee and able-bodied control subjects for both the A) 2 and B) 4 DOF pattern recognition systems.

Both transfemoral amputee and able-bodied control participants were able to control the knee and ankle of a virtual prosthesis using signals recorded from thigh muscles. Table 1 summarizes the classification accuracy and controllability results of the 2 and 4 DOF pattern recognition systems. Figure 5 shows the motion completion percentages as a function of the time. The results showed that there were no significant differences between overall classification accuracies or completion rates between subject groups or number of DOFs that the patients could control (p > 0.1, ANOVA). There was some evidence to suggest that control subjects completed motions faster than amputee subjects (p > 0.05, ANOVA). Both amputees and control subjects selected motions faster for the 2 DOF control system in comparison to the 4 DOF control system.

**Table 1:** Virtual prosthesis performance metrics

		**Knee F/E and ankle DF/PF**		**Knee F/E, femoral rotation, ankle DF/PF and ankle rotation**	
**Performance metric**		**Amputee**	**Control**	**Amputee**	**Control**
		**(n = 6)**	**(n = 6)**	**(n = 6)**	**(n = 6)**
**Classification accuracy, %**					
Overall		90.7 (6.4)	91.2 (5.5)	88.0 (3.9)	90.8 (4.7)
Hip/Knee		88.5 (10.9)	98.8 (2.0)	86.3 (9.3)	96.8 (2.7)
Ankle		88.1 (10.2)	81.4 (18.5)	86.3 (5.8)	87.2 (7.9)
**Motion selection time, s**					
Overall		0.36 (0.17)	0.38 (0.21)	0.70 (0.41)	0.58 (0.32)
Hip/Knee		0.46 (0.28)	0.33 (0.15)	0.51 (0.35)	0.57 (0.34)
Ankle		0.25 (0.14)	0.47 (0.53)	0.88 (0.85)	0.58 (0.46)
**Motion completion time, s**					
Overall		2.06 (0.56)	1.74 (0.52)	2.62 (0.67)	1.95 (0.55)
Hip/Knee		2.00 (0.85)	1.30 (0.14)	2.27 (0.63)	1.54 (0.34)
Ankle		2.14 (0.57)	2.23 (1.06)	3.00 (0.87)	2.40 (0.88)
**Motion completion percentage, %**					
Overall		97.7 (3.2)	96.3 (7.8)	90.3 (6.8)	96.5 (5.5)
Hip/Knee		100.0 (0)	100.0 (0)	95.8 (7.0)	100.0 (0)
Ankle		95.4 (6.5)	92.6 (15.6)	84.7 (11.1)	93.1 (11.1)

F/E = flexion/extension, DF/PF = dorsi-flexion/plantar flexion.

All results are presented in mean (standard deviation).

**Figure 6:**
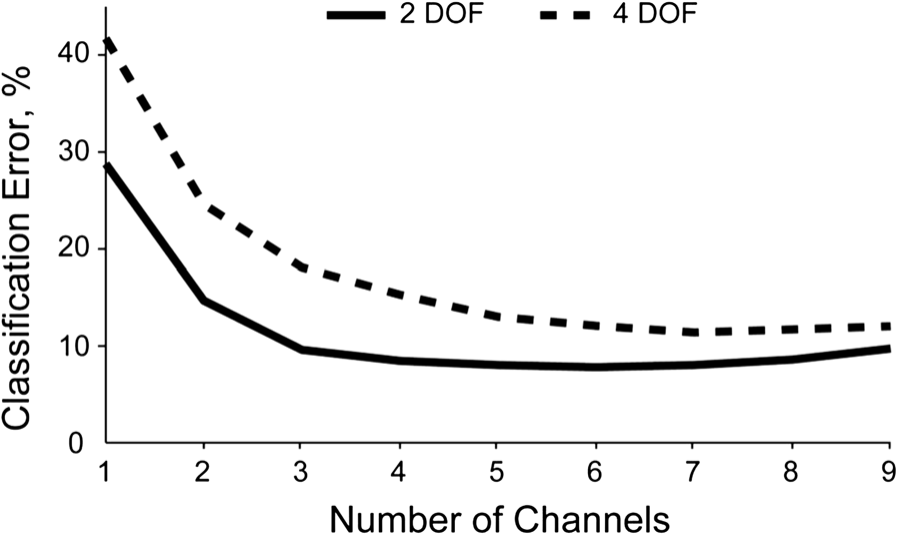
Optimal channel subset analysis results for the transfemoral amputee dataset.

An offline channel reduction analysis on the transfemoral amputee dataset showed that classification error reached a minimum when 4–6 channels were used in the pattern recognition system (Figure 6). Further analysis revealed that a selection of five muscles (semitendinosus, biceps femoris, sartorius, gracilis, and tensor fasciae latae) resulted in a system with similar performance to the optimal five-channel subset. For the 2 DOF system, classification error was 12.3% (8.8), mean (SD), using the same five muscles for each subject and 8.1% (5.6) using the optimal five-channel subset. For the 4 DOF system, classification error was 15.5% (6.9) using the same five muscles for each subject and 13.0% (6.4) using the optimal five-channel subset. Both of these classification error-rates were significantly different (p < 0.05).When only the semitendinosus and rectus femoris channels were used for the 2 DOF classifier (as described in [20]) the classification error was 18.6% (8.0) compared to 14.7% (6.9) using the optimal two-channel subset. This classification error-rate was different was significantly different (p < 0.05).

### B. Experiment 2: Non-weight-bearing Control using a Physical Prosthesis

**Figure 7:**
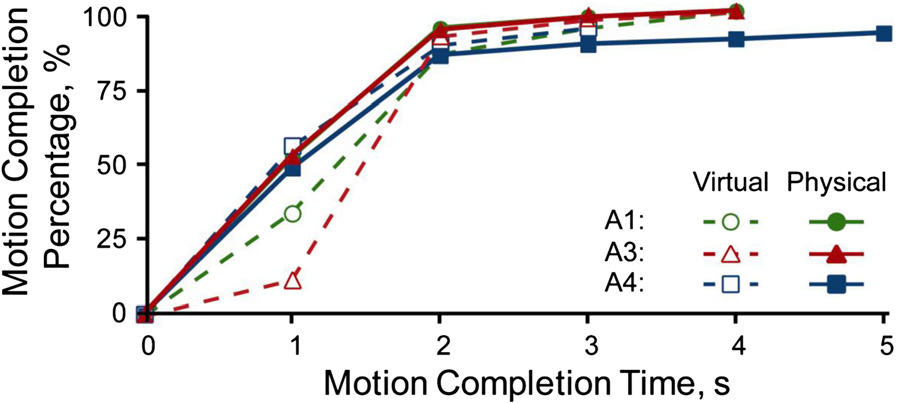
Cumulative motion completion percentage comparing performance between the physical prosthesis and the virtual prosthesis for three transfemoral amputees.

Three transfemoral amputees were able to successfully control knee flexion and extension while wearing the powered prosthesis. Averaged across subjects, the tuned impedance parameters were a stiffness, k, of 0.7 (0.1) and a damping factor, b, of 0.06 (0.02). Subjects performed slightly better with the physical prosthesis in comparison to using only the virtual environment (Table 2, Figure 7). Importantly, the pattern recognition system could still reliably decode ankle motions when the knee joint was repositioned at a 45 degree angle.

**Table 2:** Comparison of physical and virtual prosthesis performance metrics

**Performance metric**	**Virtual prosthesis**	**Physical prosthesis**
**Classification accuracy, %**		
Overall	90.7 (3.2)	92.0 (5.0)
Knee	92.7 (1.6)	93.0 (2.2)
Ankle	85.0 (7.4)	87.5 (10.2)
**Completion time, s**		
Overall	1.32 (0.1)	1.26 (0.1)
Knee	1.23 (0.3)	1.11 (0.1)
Ankle (all)	1.40 (0.3)	1.32 (0.1)
Ankle (knee at 90 deg)	n/a	1.37 (0.3)
Ankle (knee at 45 deg)	n/a	1.31 (0.2)
**Completion rate, %**		
Overall	98.2 (3.2)	96.3 (4.3)
Knee	100.0 (0)	100.0 (0)
Ankle (all)	96.3 (6.4)	97.2 (4.8)
Ankle (knee at 90 deg)	n/a	100.0 (0)
Ankle (knee at 45 deg)	n/a	88.9 (12.8)

All results are presented in mean (standard deviation).

## Discussion

Accurate classification and controllability of knee and hip movements was expected because the EMG signals were recorded from physiologically appropriate residual limb muscles. Accurate classification and controllability of ankle motions was unexpected; the muscles that control the ankle are located below the knee and were lost as a result of amputation. Nonetheless, transfemoral amputees were generating distinct and repeatable co-activity patterns even after an average time of 26.3 (17.7) years post-amputation. These patterns were properly interpreted by the pattern recognition system. This is analogous to recognizing subtle differences in hand grasp patterns using only the extrinsic forearm muscles of transradial amputees [35].

There was no significant difference (p > 0.1) between the performances of the able-bodied control participants and the amputee participants in terms of overall classification accuracy or motion completion rate for the 2 and 4 DOF pattern recognition systems. We believe this is primarily because only above knee muscles were sampled from the control subjects; however, we did expect that the control participants would perform slightly better than the amputee participants because visual and proprioceptive feedback make it easier to perform repeatable movements. There was some evidence (but not statistically significant) to suggest that control subjects completed motions faster than amputee subjects, but the differences in time were very small (<0.5 s). We believe that the therapist’s instructions prior to data collection helped to compensate for the lack of feedback available to the amputee participants. This is consistent with upper-limb pattern recognition work in which we have noted that patient training helps to improve control, especially for novice users [36,37]. Both amputee patients and control subjects took longer to select motions for the 4 DOF control system. We believe that the patients had to think longer about which motion they were being asked to control in the 4 DOF test.

Previous studies have demonstrated impressive offline classification accuracies [28,29] or position tracking of knee movements [30] with healthy control subjects. The capability for transfemoral amputees to control knee movements in a virtual environment has also been investigated but the patients were not wearing the prosthesis [26]. In the current study, only 2 DOFs were evaluated with the physical prosthesis. Saggital plane movements of the knee and ankle were selected because there are currently 2 DOF mechanically powered legs under development. Although these results are preliminary, they are promising. All subjects were able to reliably to control the knee in real time. Furthermore, the pattern recognition system properly interpreted ankle commands when the prosthesis was repositioned to a 45 degree angle, suspending freely in space. This suggests that myoelectric signal changes resulting from dynamic loading on the socket do not degrade lower limb pattern recognition performance. Further testing with additional amputees is required to see if this result can be generalized across subjects. It also should be noted that only changes in the knee angle were tested and not changes in the position of the residual limb.

Proportional control estimates of knee velocity were not incorporated into the control system, and the parameters of the myoelectric impedance controller were adjusted empirically by the experimenter. Proportional control signals may be added by taking a simple average of EMG amplitudes [38] or by using a weighted average of EMG amplitudes determined by principle component analysis [22]. Smoother kinematic profiles may be obtained by optimizing the selection of the impedance parameters—the objective of ongoing research.

The channel subset analysis showed that only 4 channels of EMG data are required to achieve low classification errors when only 2 DOFs are controlled. Using fewer EMG channels simplifies the socket modification required to collect the signals and reduces the chances of a wire failure. The optimal channel subset was slightly different across the six transfemoral amputees for the 2 and 4 DOF systems. Selecting the most common five muscles, including the semitendiosus, biceps femoris, sartorius, gracilis and tensor fasciae latae, resulted in a system with only slight increases in classification error above the optimal five-channel set. The performance obtained when using only the semitendinosus and rectus femoris channels was significantly different than using the optimal 4–6 channel subsets. This is somewhat to be expected since Ha et al. only investigated 1 DOF, a much more simple system than the current study’s 2 and 4 DOF systems.

The benefits of intuitive non-weight-bearing control are that it allows the patient to reposition the limb for increased comfort or in preparation for a transition. These pattern recognition systems are quite insensitive to muscle crosstalk [33]; however they are sensitive to electrode shifts with respect to the residual limb [39], changes in residual limb position [40], and changes in electrode impedance. These issues may be mitigated by collecting more training data or altering the electrode configuration [41]. The proposed algorithm was only developed for use during non-weight-bearing situations. While this type of control is important, our ultimate goal is to investigate the benefits of using EMG signals to supplement mechanical sensor data in weight-bearing situations. A different signal processing approach, such as making use of neuromuscular mechanical sensor fusion [25], is necessary for weight-bearing situations, especially when the patient is ambulating, as there may be large movement artifacts on the signal or the electrodes may even lose contact with the socket.

## Conclusion

This work demonstrates the feasibility of extracting neural information from above knee muscles that is suitable for controlling both knee and ankle movements of powered lower limb prostheses during non-weight bearing activates. To our knowledge, this is the first time EMG signals from the residual muscles of transfemoral amputees have been used to directly control both knee and ankle movements. Current work is focusing on modifying the powered knee prosthesis control system so that neural information can be used during weight-bearing activities.

## Appendix A

### Time-domain feature extraction

The time-domain statistics proposed by Hudgins *et al.*[42] consisted of the MAV, the number of zero crossings, the number of slope sign changes, and the waveform length. These four statistics were computed for each EMG input channel and are concatenated to form a feature vector.

### *Mean absolute value*

An estimate of the mean absolute value of the signal, *x*(*t*), in analysis window *i* with *N* samples is given by:

$${\bar{X}}_{i}=\frac{1}{N}\overset{N}{\underset{n}{\sum }}\left|x\left(n\right)\right|,i=\left(1,\ldots ,I\right)$$

Where *x*(*n*) is the n^th^ sample in analysis window *i*, and *I* is the total number of analysis windows over the entire sampled signal.

### *Number of zero crossings*

A simple frequency measure can be obtained by counting the number of times the waveform *x*(*t*) crosses zero. To reduce the number of noise-induced zero crossings, a threshold value of ϵ is included in the calculation. Given two consecutive values of the signal, x_n_ and x_n+1_, the zero crossing count is incremented if:

$$\left\{x_{n}>0\;\mathrm{and}\;x_{n+1}<0\right\}\;\mathrm{or}$$

$$\left\{x_{n}<0\;\mathrm{and}\;x_{n+1}>0\right\}\;\mathrm{and}$$

$$\begin{array}{l} \left|x_{n}-x_{n+1}\right|\geq \epsilon \end{array}$$

The value of ϵ is dependent on the system noise and needs to be selected appropriately by examining the noise levels of the data.

### *Slope sign changes*

Another feature which may provide a measure of frequency content is the number of times the slope of the waveform *x*(*t*) changes sign. Once again a threshold value *ϵ* must be used to reduce noise induced slope sign changes. Given three consecutive values of the signal *x*_n-1_, *x*_*n*_, and *x*_*n*+1_ the slope sign change count is incremented if

$$\left\{x_{n}>x_{n-1}\;\mathrm{and}\;x_{n}>x_{n+1}\right\}\;\mathrm{or}\;\left\{x_{n}<x_{n-1}\mathrm{and}\;x_{n}<x_{n+1}\right\}$$

And

$$\left\{\left|x_{n}-x_{n+1}\right|\geq \epsilon \right\}\;\mathrm{or}\;\{|x_{n}-x_{n-1}|\geq \epsilon \;$$

The value of ϵ is once again dependent on the noise and should be selected appropriately.

### *Waveform length*

This feature provides information on the complexity of the waveform in each analysis window. It is the cumulative length of the waveform defined as:

$$l_{i}=\overset{N}{\underset{n=1}{\sum }}\left|\Delta x_{n}\right|$$

Where Δ*x*_*n*_ = *x*_*n*_-*x*_*n*-1,_ is the difference between consecutive signal samples. The resultant value provides a measure of waveform amplitude, frequency, and duration all within a single parameter.

## Linear discriminant analysis (LDA)

A linear discriminant classifier is a simplified implementation of a Bayesian statistical classifier. The Bayes classification rule states: assign the *N*-length pattern **x** to the class,*C*_*i*_ so that the following inequality is satisfied

$$P\left(C_{i}|x\right)>P\left(C_{j}|x\right),\mathrm{for}\;i\neq j$$

However, these *a posteriori* probabilities cannot be directly measured. Instead, Bayes’ Theorem

$$P\left(x|C_{i}\right)=P\left(C_{i}\right)p\left(x|C_{i}\right)=p\left(x\right)P\left(C_{i}|x\right)$$

can be used to provide the solution by deriving the *a posteriori* probabilities from estimates of the *a priori* probabilities

$$P\left(C_{i}|x\right)=\frac{P\left(C_{i}\right)p\left(x|C_{i}\right)}{P\left(x\right)}$$

Where P(**x**|*C*_*i*_) is the probability density function for the samples within the i^th^ class and *p*(**x**) is the probability density function of the input space and is a constant over all the classes. It is usually assumed that the probabilities of the output classes *P*(*C*_*i*_), are equal. Now application of Bayes’ classification rule essentially becomes evaluating

$$d_{i}\left(x\right)=P\left(C_{i}\right)p\left(x|C_{i}\right)$$

for each of the *M* classes and choosing the maximum value.

The LDA implementation simplifies the Bayesian classifier by assuming that all probability density functions are Gaussian. The multivariate Gaussian probability density function for *M* classes of patterns can be expressed as:

$$p\left(x|C_{i}\right)=\frac{1}{2\pi^{\frac{N}{2}}{\left|C_{i}\right|}^{\frac{1}{2}}}\exp\left[-\frac{1}{2}{\left(x-m_{i}\right)}^{T}C_{i}^{-1}\left(x-m_{i}\right)\right],i=1,\ldots ,M$$

Where **m**_*i*_ is the *N* length mean vector for the i^th^ class and $C_{i}$ is the *N* x *N* covariance matrix for the i^th^ class. Now,

$$d_{i}\left(x\right)=\frac{P\left(C_{i}\right)}{2\pi^{\frac{N}{2}}{\left|C_{i}\right|}^{\frac{1}{2}}}\exp\left[-\frac{1}{2}{\left(x-m_{i}\right)}^{T}C_{i}^{-1}\left(x-m_{i}\right)\right]$$

Expressing this value in the natural logarithm form and canceling constant terms yields

$$d_{i}\left(x\right)=\ln\left(P\left(C_{i}\right)\right)-\ln\left(C_{i}\right)-{\left(x-m_{i}\right)}^{T}C_{i}^{-1}\left(x-m_{i}\right)$$

Furthermore, by assuming that all the covariance matrices are equal, the set of discriminant functions becomes

$$d_{i}\left(x\right)=\ln\left(P\left(C_{i}\right)\right)+x^{T}C^{-1}m_{i}-\frac{1}{2}m_{i}^{T}C^{-1}m_{i}$$

The classification now becomes a problem of *M* equations and *N* unknowns, where *M* is the number of classes and *N* is the length of the feature vector.*d*(**x**) could also be expressed in terms of *M* x *N* weight matrix and a *M* x 1 offset array

$$W=C^{-1}m_{i}$$

$$B=-\frac{1}{2}m_{i}^{T}C^{-1}m_{i}+\ln\left(P\left(C_{i}\right)\right)$$

$$d\left(x\right)=x^{T}W+B$$

After the weights and the offset have been calculated from an appropriate set of training data, feed-forward classification using a LDA is computationally simple.

## Competing interests

The authors declare that they have no competing interests.

## Authors’ contributions

LH and AS were responsible for study design, data analysis and manuscript preparation. RL and SF were responsible for data collection and critical review of the manuscript. TK was responsible for study design, obtaining funding, and critical review of the manuscript. All authors read and approved the final manuscript.
